# Changes in the Gut Microbiota May Affect the Clinical Efficacy of Oral Anticoagulants

**DOI:** 10.3389/fphar.2022.860237

**Published:** 2022-03-25

**Authors:** Wenjun Chen, Jiafen Qian, Jinglan Fu, Tingting Wu, Meina Lv, Shaojun Jiang, Jinhua Zhang

**Affiliations:** ^1^ Department of Pharmacy, Fujian Medical University Union Hospital, Fuzhou, China; ^2^ College of Pharmacy, Fujian Medical University, Fuzhou, China

**Keywords:** gut microbiota, oral anticoagulants, bioavailability, liver drug enzymes, P-gp

## Abstract

The mechanism underlying large individual differences in the response to oral anticoagulants has not been fully clarified, and the influence of the intestinal microbiome on exogenous drug metabolism has gradually become an area of increased research interest. However, there has been no research into the influence of the gut microbiota on the pharmacokinetics of oral anticoagulants. Therefore, our study is the first to investigate the effect of the intestinal flora on oral anticoagulant metabolism and the associated mechanism. Antibiotics affected the diversity and abundance of the intestinal flora. Compared with the control group, the bioavailability of warfarin and rivaroxaban were significantly increased in the amoxicillin-treated group, whereas the bioavailability of dabigatran increased and subsequently decreased. Compared with the control group, the expression of P-glycoprotein (P-gp), CYP1A2, CYP2C9, CYP3A4, and nuclear receptor, PXR, were altered in the amoxicillin -treated groups. This trend was consistent with the pharmacokinetic results. Changes in the intestinal flora can affect the expression of liver drug enzymes and P-gp, as well as affect the transport and metabolism of oral anticoagulants (e.g., warfarin, dabigatracin, and rivaroxaban), leading to differences in the efficacy of oral anticoagulants. This study revealed a novel mechanism for influencing individual differences in the treatment efficacy of oral anticoagulants.

## Introduction

Anticoagulant therapy (i.e., oral anticoagulants) represents the primarily form of treatment for thromboembolic diseases. Oral anticoagulants, including traditional oral anticoagulants and direct oral anticoagulants, are drugs that inhibit blood coagulation by inhibiting or blocking various pathways associated with the body’s physiological blood coagulation mechanism. Warfarin exerts an anticoagulant role by inhibiting vitamin K, and has been widely used for the prevention of atrial fibrillation, thrombosis following mechanical heart valve replacement, and the treatment of venous thromboembolism (Zhang et al., 2014; Kearon et al., 2016). Direct oral anticoagulants (DOACs), which include coagulation factor inhibitors (e.g., rivaroxaban, apishaban, and idoxaban) and thrombin inhibitors (e.g., dabigatran), have gradually begun to replace warfarin for the treatment of atrial fibrillation, deep vein thrombosis, and pulmonary embolism. This is primarily because DOACs have more predictable pharmacokinetic and pharmacodynamic indicators compared to warfarin (Heidbuchel et al., 2015). Thus, with the increasing morbidity and mortality of venous thromboembolic diseases, the rational application of oral anticoagulants is of great significance.

However, clinical trials and practice of oral anticoagulants have shown substantial individual differences in treatment efficacy and the occurrence of drug interactions, which has resulted in less than 50% of patients receiving an ideal therapeutic effect; about 40% of patients are in the sub-effective range, and about 20% of patients are beyond the effective treatment range (Lu et al., 2016). In addition, the efficacy of oral anticoagulants is closely related to their adverse reactions. According to statistics, the frequency of warfarin bleeding events and severe bleeding events remains as high as 7.5–16.5% and 1.3–4.2%, respectively (Bi et al., 2018). Although DOACs have a lower incidence of adverse events than warfarin, bleeding remains a serious complication, with a 3.9% frequency of bleeding events (Kwon et al., 2018). Therefore, elucidation of the factors that influence the difference in the clinical efficacy of oral anticoagulants and their individual application have been the focus of attention (Mcmilln et al., 2011; Pellagatti et al., 2017). Differences in the clinical efficacy and adverse reactions of oral anticoagulants often depend on changes in the expression levels and function of the enzymes involved in drug metabolism and transporter proteins. The treatment efficacy of warfarin in the body is mainly affected by the expression of the liver drug enzymes, CYP2C9, CYP3A4, and CYP1A2. Moreover, dabigatrine ester is the substrate of the efflux transporter P-glycoprotein (P-glycoprotein), which is not metabolized by liver drug enzymes. Rivaroxaban is the substrate of P-glycoprotein, which is metabolized by the liver drug enzymes, CYP1A2 and CYP3A4, and is jointly influenced by drug transporter and drug metabolizing enzymes (Gnoth et al., 2011; Kishimoto et al., 2013; Zhang et al., 2013; Hodin et al., 2017). During the process of seeking personalized treatment with oral anticoagulants, most studies have focused on how an individual’s genome controls the body’s response to the drug; however, there is growing evidence that a person’s unique microbiome, characterized by the amount and phenotype of bacteria and other microbes living in their bodies, is the key to determining whether a drug will be effective for their condition.

With the development of high-throughput, low-cost sequencing and metabolomics methods, studies investigating the influence of the gut microbiome and its metabolic activity on the metabolism of exogenous drugs has gradually become a new research focus. The study by Jeong et al. (Kang et al., 2014) found that changes in the gut microbiota could affect the pharmacokinetic parameters of baicalin, suggesting that gut microbiota may play a key role in the oral pharmacokinetic dynamics of baicalin. Another study found that the bioavailability of amlodipine in rats was significantly improved after 3 days of oral ampicillin treatment (Yoo et al., 2016). And *in vitro* culture experiments have also confirmed that the bacterial flora altered by antibiotics reduced the metabolism of amlodipine. In summary, it has been confirmed that the microbiome can significantly affect the metabolic process of exogenous drugs in a variety of metabolic pathways, including the expression of liver drug enzymes, abc-like transporters, and the functional differences of metabolites. However, there has been no research into the influence of the gut microbiota on the pharmacokinetics of oral anticoagulants.

Therefore, this study successfully established a model of gut dysbiosis by administering amoxicillin to rats, and is the first to investigate the influence of gut microbiota on the pharmacokinetic parameters of oral anticoagulants. The mechanism by which intestinal microbiota affects the bioavailability of oral anticoagulants was studied from the perspective of liver metabolism and intestinal absorption. The results of this study will provide important data for the study of the factors and mechanisms that have an impact on the differences in the clinical efficacy of oral anticoagulants, as well as help promote more comprehensive personalized treatment of oral anticoagulants.

## Materials and Methods

### Ethics Approval

Animal experiments were approved and performed in accordance with the guidelines of the Institutional Animal Care and Use Committee of Fujian Medical University (approval No. FJMUIACUC 2020-0049).

### Animals

Animal experiments were approved and performed in accordance with the guidelines of the Institutional Animal Care and Use Committee of Fujian Medical University (approval No. FJMUIACUC 2020-0049). Six-week-old male Sprague-Dawley rats were purchased from Shanghai SLAC Laboratory Animal Co., Ltd.) (Shanghai, China) and housed at 22°C ± 2°C in a standard cage within a specific pathogen-free facility on a 12:12-h light:dark cycle. The SD rats were divided into three groups: 1) control group; 2) 3 days antibiotic treatment group; and the 7 days antibiotic treatment group. Water and chow were available ad libitum. Amoxicillin was dissolved in ultrapure water, and was administered by gavage to the rats every day at 8:00, 16:00, and 23:00, at a dose of 90 mg/kg (body weight). Before and after modeling, fecal samples were collected for a subsequent gut microbiota analysis. After successful modeling, the following oral anticoagulants were administered orally: warfarin sodium (0.2 mg. Kg-1, Qilu Pharmaceutical Co., LTD. China), dabigatrol (20 mg/kg, Boehringer Ingelheim Pharma GmbH & Co. Kg, Germany), and rivaroxaban (2.6 mg/kg, Bayer Corporation, Germany). Blood samples were collected at pre-established blood sampling time points for subsequent pharmacokinetic studies and ileum and liver samples were collected for subsequent studies after anesthesia.

### Analysis of Gut Microbiota

Fresh rat feces were collected, quickly placed in liquid nitrogen, and subsequently stored at −80°C. DNA was extracted from 0.25 g of feces using the E.Z.N.ATM Mag-Bind Soil DNA Kit (OMEGA, United States). To assess the composition of the microbial community, the hypervariable V3-V4 regions of 16S rRNA genes were amplified by PCR (5 min at 95°C, followed by 20 cycles of 30 s at 94°C, 20 s at 55°C, 30 s at 72°C, and a final 5 min at 72°C) using the primers for 341F and 805R (341F, 5′-CCTAYGGGRBGCASCAG-3′; 805R, 5-GACTACHVGGGTATCTAATCC-3′). Following further PCR purification, the eluted DNA products were quantified using a Qubit 3.0 DNA Kit assay (Life, United States) and pooled in equal proportion into a single library. Purified amplicons were pooled for sequencing on an Illumina MiSeq platform according to the manufacturer’s instructions. QIIME (version 1.17) was used to process raw fastq files. High-quality reads were obtained after quality filtering and the samples were clustered into operational taxonomic units (OTUs) at a 97% similarity cut-off using UPARSE.

### Analysis of Oral Anticoagulant Levels in Blood

#### Blood Concentration of Warfarin Was Determined by HPLC

The experimental methods were performed based on previous studies (Chu et al., 2011). The preparation of warfarin was dissolved in ultrapure water (0.2 mg·kg^−1^). Blood was collected at 0, 0.5, 1, 2, 4, 8, 12, 24, 36, 48, and 72 h after administration. Chromatographic column: SinoChrom ODS-BP C18 (4.6 × 150 mm, 5 μm); mobile phase: acetonitrile/water/formic acid (50: 50: 0.2); flow velocity: 0.8 ml/min; column temperature: 30°C; ultraviolet (UV) detection wavelength: 305 nm; injection quantity: 40 μl. Chromatograms were recorded and the peak areas were quantified using the external standard method.

#### Blood Concentration of Dabigatran and Rivaroxaban Were Determined by LC-MS/MS

The experimental methods were performed based on previous studies (Baldelli et al., 2016). Dabigatrol mesylate was suspended with 0.5% carboxymethyl cellulose sodium (20 mg/kg). Blood was collected at 0, 0.25, 0.5, 0.75, 1, 1.5, 2, 3, 4, 6, 8, 10, 24, and 60 h after administration. Rivaroxaban was prepared with a 0.5% methyl cellulose aqueous solution (2.6 mg/kg). Blood was collected at 0, 0.17, 0.33, 0.5, 0.75, 1, 1.5, 2, 3, 4, 6, 8, 12, and 24 h after administration.

Chromatographic conditions: XBridge^®^ C18 column (100 × 2.1 mm, 3.5 μm); mobile phase: 10 mmol/L ammonium formate (A phase)-methanol (B phase), gradient elution; flow velocity: 0.3 ml/min; column temperature: 30°C; injection quantity: 10 μl.

Mass spectrometry conditions: Electrospray ionization source (ESI) positive ion mode scanning was performed. A multiple response monitoring model (MRM) was used for quantitative analysis. Other major mass spectral parameters: atomizer pressure: 30 psi. dry gas velocity: 10.0 L/min; ion source temperature: 350°C; capillary: 4,000 V; scanning time: 60 ms.

### Hepatic Metabolic Enzyme and P-Glycoprotein Protein Expression

Liver and terminal ileum tissue samples were homogenized in RIPA (Beyotime Biotechnology, China), respectively. The total protein concentrations of the homogenates were measured with BCA reagent (BOSTER, China). An equal amount of protein was subjected to sodium dodecyl sulfate polyacrylamide gel electrophoresis (SDS-PAGE) and transferred to polyvinylidene difluoride membranes by electroblotting. After blocking, the membranes were incubated with primary antibodies directed against human CYP1A2, CYP3A4, CYP2C9, P-glycoprotein, and GAPDH (Abcam, UK). Software ImageJ was used to analyze the Gray scale of the obtained images:

Gray value of each protein = gray value of target protein/gray value of internal reference protein.

### Hepatic Metabolic Enzyme mRNA Expression

RNA was extracted from the liver and terminal ileum tissues using TRIzol reagent (CWBIO, China), and cDNA was synthesized by reverse transcription using HiScript II qRT SuperMix II (Vazyme, China). SYBR Green PCR Master Mix (Lifeint, China) was used for PCR quantification. A repeat analysis was performed on each sample. PCR amplification was used to collect the stored reaction information after the reaction. The 2^-△△Ct^ method was used to calculate the relative expression of each the target gene.

### Statistical Analysis

SPSS 22.0 statistical software was used for the statistical analysis. The data were expressed as mean ± SEM. Comparison of the experimental data between groups was performed by the *t* test and one-way ANOVA, or non-parametric test. *p* < 0.05 was considered statistically significant.

## Results

### Effects of Amoxicillin on Gut Microbiota

As shown in Table 1, the OTUs number was similar before modeling in the control group before modeling (Group A), 3 days amoxicillin treatment group before modeling (Group B), 7 days amoxicillin treatment group before modeling (Group C), and the control group after modeling (Group D), and significantly higher than the 3 days amoxicillin treatment group after modeling (Group E, 249 ± 74) and 7 days amoxicillin treatment group after modeling (Group F, 397 ± 38). The results showed that the intestinal flora composition in the rats was significantly reduced. Moreover, the Chao and ACE indexes of the six groups were: A > B > D > C > F > E, indicating that the intestinal bacterial community richness was reduced in each of the six groups. For community diversity, groups A, B, C, and D had a similar Shannon index, which was higher than that of groups E and F for 7 days. The results showed that amoxicillin treatment could reduce the α-diversity of the gut microbiota in the rats.

**TABLE 1:** Comparison of the abundance and diversity of intestinal microflora of the rats before and after modeling.

Sample_ID	Seq_num	OTU_num	Shannon_index	Simpson	ACE_index	Chao1_index	Coverage
A	51,926	1,316	4.3558	0.0576	2,490.1789	1,988.6411	0.9899
B	50,232	1,358	4.6035	0.0331	2,117.4247	1,898.9856	0.9900
C	43,456	1,210	4.6534	0.0338	1,835.5451	1,697.1137	0.9899
D	49,831	1,231	4.2826	0.0561	2,072.8923	1,740.6449	0.9906
E	58,117	249	1.2769	0.4210	473.5920	363.7650	0.9980
F	47,585	397	3.1780	0.1086	569.9020	506.1724	0.9975

Note: Control group before modeling (Group A), the antibiotic treated for 3 days group before modeling (Group B), the antibiotic treated for 7 days group before modeling (Group C), Control group after modeling (Group D), the antibiotic treated for 3 days group after modeling (Group E), and the antibiotic treated for 7 days group after modeling (Group F).

Sample_ID: Sample name Seq_num: Number of good reads of the sample OTU_num: Number of OTU, obtained from the sample clustering. The remaining five columns are the respective values of the five Alpha indexes.

**FIGURE 1:**
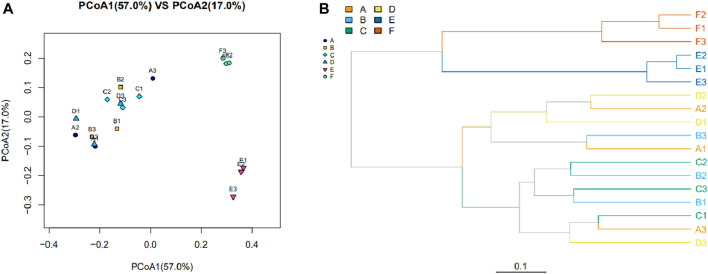
The bacterial community and composition between samples were evaluated with a Beta diversity analysis. **(A)** Principal coordinate analysis based on weighted UniFrac. Each point represents a sample, with points of the same color derived from the same group. The closer the distance between the two points, the smaller the community composition difference. **(B)** Cluster analysis of bacterial community composition among different samples based on the weighted UniFrac. The length of the branches represents the distance between the samples; the closer the samples are on the branches, the more closely related the sample are to each other. The branches of the same color are from the same group.

As shown in Figure 1, the β-diversity in the structure and composition of the intestinal microbiome was assessed. The bacterial communities of the samples from groups A, B, C, and D were clustered together without obvious differentiation, indicating a similar bacterial flora structure and composition between the four groups. The bacterial communities in the samples from groups E and F were distinct from the other samples and clustered separately. This finding indicated that the gut microbiota changed regarding the community structure and composition following amoxicillin treatment, which was different from that of the first four groups (Figure 1A). Similar results were found in the hierarchical clustering analysis based on UniFrac (Figure 1B).

### Effects of Antibiotics on the Distribution of Dominant Flora

**FIGURE 2:**
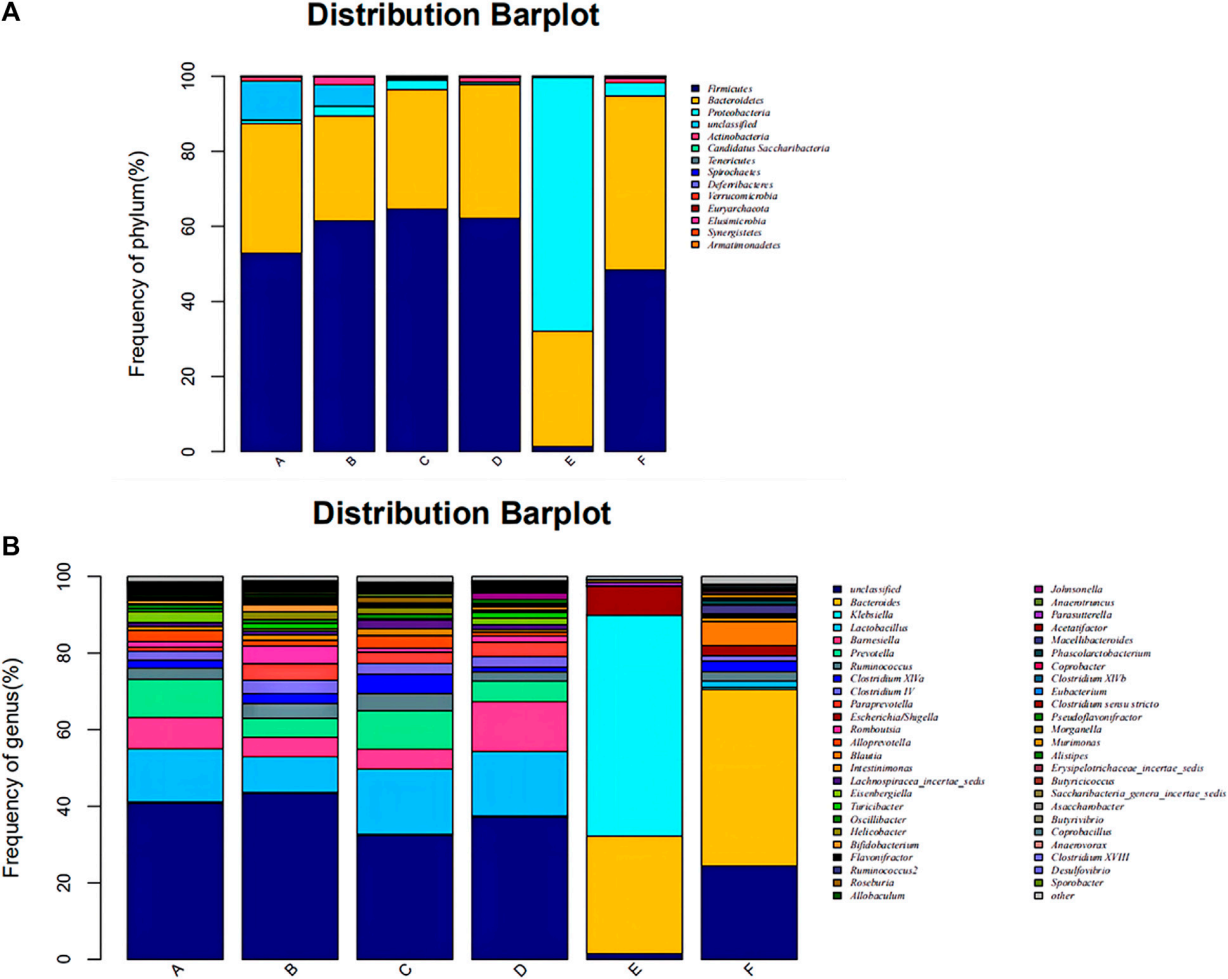
Composition and distribution of the intestinal flora in each group at the phylum and genus level. Composition and distribution of the intestinal flora in each group at **(A)** the phylum level and **(B)** the genus level. The abscissa is arranged according to the sample name, each bar chart represents a sample, and the taxon is distinguished by color. The ordinate represents the relative abundance of each taxon, and the longer the column, the higher the phase abundance of the taxon in the corresponding sample. Control group before modeling (Group A), antibiotic treated for 3 days group before modeling (Group B), the antibiotic treated for 3 days group before modeling (Group C), Control group after modeling (Group D), the antibiotic treated for 3 days group after modeling (Group E), and the antibiotic treated for 7 days group after modeling (Group F).

**FIGURE 3:**
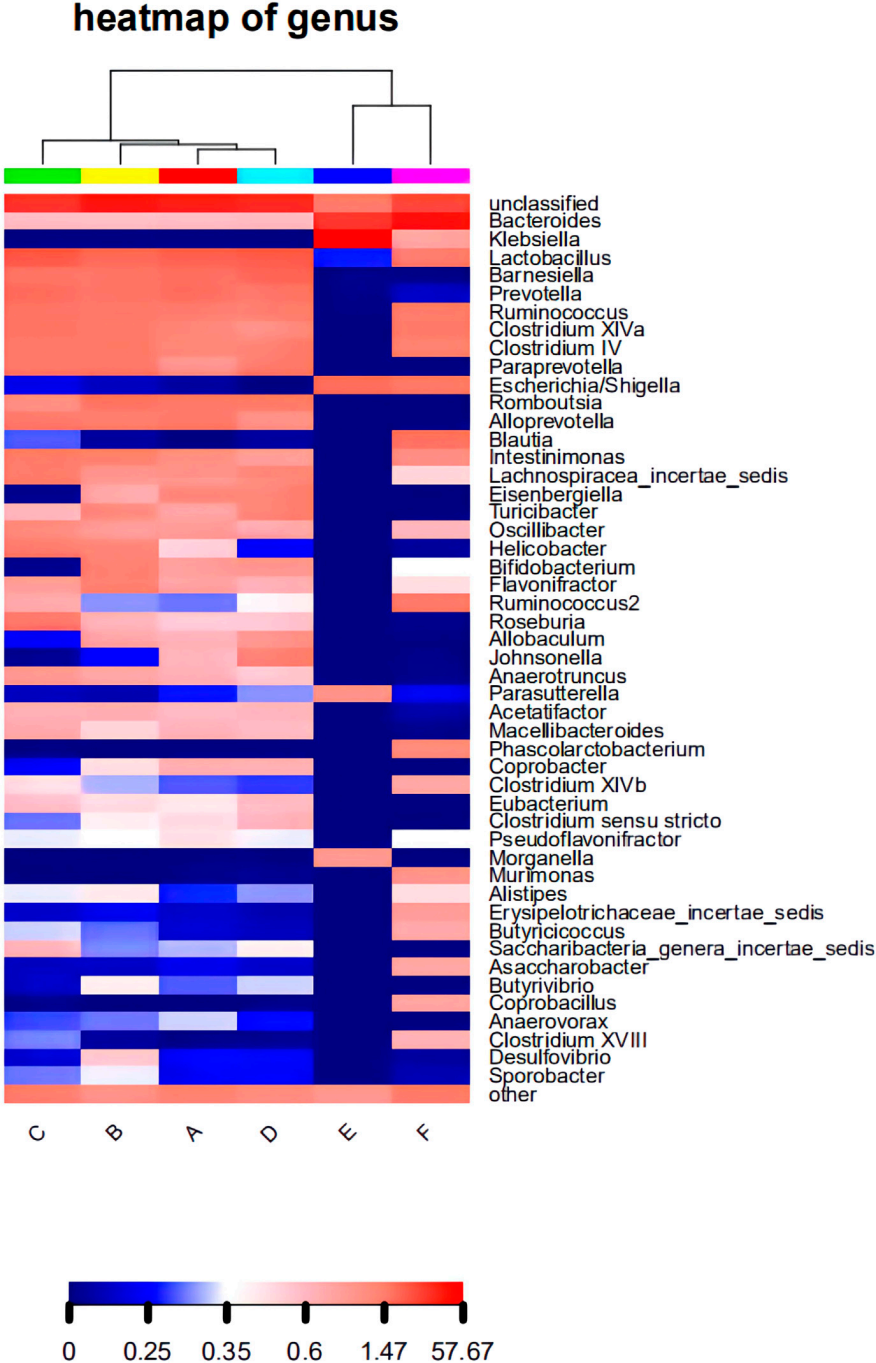
The level of abundance of the top 50 genera in each group of samples. The samples were first clustered according to their similarity, and subsequently arranged laterally according to the clustering results. Similarly, taxa are also clustered according to the distribution similarity of each taxon in different samples, and arranged vertically according to the clustering results. In the figure, red represents the genus with a higher abundance in the corresponding sample, and blue represents the genus with a lower abundance.

The composition of the bacterial community in each sample at the level of phylum and genus classification is shown in Figure 2. Figure 2A shows that the composition and abundance of the intestinal bacterial community of each group before and after modeling exhibited high similarities at the phylum classification level, which primarily included Firmicutes, *Bacteroides*, Proteobacteria, and Actinomycetes. Under amoxicillin treatment, the distribution ratio of each dominant phyla changed, among which, the 3 days amoxicillin treatment group after modeling (Group E) exhibited the greatest change. The proportion of Firmicutes and Bacteroidetes decreased, whereas the proportion of Proteobacteria increased and became the most dominant bacterial phylum. As shown in Figure 2B, the intestinal bacteria of each group had similar community structure composition at the genus classification level before and after modeling. The proportion of community flora were similar in the control group before modeling (Group A), 3 days amoxicillin treatment group before modeling (Group B), 7 days amoxicillin treatment group before modeling (Group C) and the control group after modeling (Group D). After amoxicillin treatment, the distribution and proportion of the dominant bacteria changed, among which the distribution ratio of the 3 days amoxicillin treatment group underwent the greatest change. In addition, similar results were obtained in the heatmap constructed according to the abundance level of the top 50 genera in each sample (Figure 3). These results showed that amoxicillin treatment changed the structure of the intestinal flora in rats, and the 3 days amoxicillin treatment group had the greatest influence on the intestinal flora.

### Pharmacokinetics of Oral Anticoagulants in Rats With a Gut Dysbiosis

**FIGURE 4:**
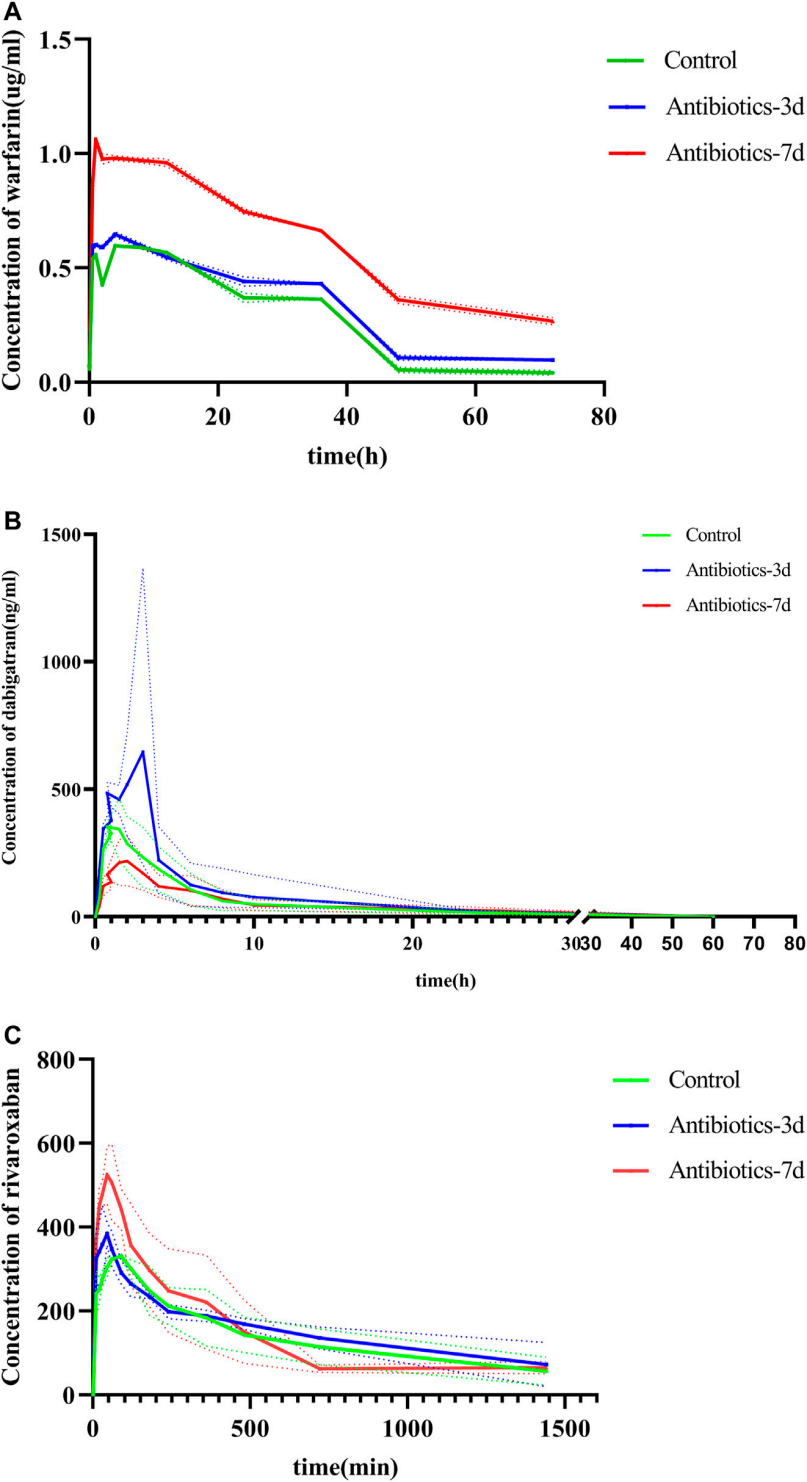
The concentration of oral anticoagulants in the plasma over time. **(A)** Warfarin, **(B)** dabigatran, and **(C)** rivaroxaban. Comparison of drug-time curves among the control group, antibiotic treated for 3 days group, and antibiotic treated for 7 days group. Note: Different colors represent the different groups, broken lines are measured values, dashed lines are errors, and curves are fitted by measured data.

After successfully establishing of a rat model of gut dysbiosis, the effects of a gut dysbiosis on the pharmacokinetic processes associated with oral anticoagulants were further studied, and the results are shown in Figure 4. As shown in Figure 4A and Table 2, the bioavailability of warfarin in the amoxicillin-treated group was increased compared with that of the control group. And the bioavailability of warfarin in the 7 days amoxicillin treatment group showed the greatest increase, at 115% (*p* < 0.05). In addition, the gut dysbiosis also reduced the clearance rate of warfarin, extended the elimination half-life, reduced the time to peak concentration, and increased the peak concentration (Table 2). As shown in Figure 4B and Table 3, compared with the control group, the maximum increase in bioavailability of dabigatran was observed in the rats treated with amoxicillin for 3 days, which increased by 53% (*p* < 0.05). However, the bioavailability of dabigatran was significantly decreased by 11% in the 7 days amoxicillin treatment group. As shown in Figure 4C and Table 4, compared with the control group, the bioavailability of rivaroxaban increased in rats in the amoxicillin treatment groups, with an increase of 10% observed in the 3 days amoxicillin treatment group. Similarly, gut dysbiosis also affected the clearance rate, elimination half-life, and peak concentration time of dabigatran and rivaroxaban (Tables 3 and 4).

**TABLE 2:** Comparison of the pharmacokinetic parameters of Warfarin (n = 3).

Parameter	Control	Antibiotic-3days	Antibiotic-7days
AUC(0-t)/mg/L*h	20.301 ± 0.494	23.877 ± 0.58	43.821 ± 0.57**
AUC(0-∞)/mg/L*h	21.217 ± 0.784	27.193 ± 0.717*	56.34 ± 1.472**
MRT (0-t)/h	20.854 ± 0.573	23.865 ± 0.246	26.971 ± 0.181*
t1/2z/h	15.468 ± 1.47	23.383 ± 0.85*	32.454 ± 1.751**
Tmax/h	4	4	1*
CLz/F/L/h/kg	0.009 ± 0.001	0.007 ± 0.001	0.004 ± 0.001*
Vz/F/L/kg	0.21 ± 0.013	0.248 ± 0.009	0.166 ± 0.006*
Cmax/mg/L	0.607 ± 0.006	0.647 ± 0.012	1.06 ± 0.006**

Note: *means *p* < 0.05; **means *p* < 0.01.

**TABLE 3:** Comparison of the pharmacokinetic parameters of Dabigatran (n = 3).

Parameter	Control	Antibiotic-3days	Antibiotic-7days
AUC(0-t)/mg/L*h	2.350 ± 0.619	3.609 ± 2.139*	2.101 ± 0.347
AUC(0-∞)/mg/L*h	2.351 ± 0.619	3.612 ± 2.143*	2.102 ± 0.347
MRT (0-t)/h	8.505 ± 2.325	7.633 ± 0.501	11.472 ± 3.193
t1/2z/h	6.008 ± 0.256	5.816 ± 1.216	5.688 ± 0.631
Tmax/h	1.25 ± 0.433	2 ± 1.000	2
CLz/F/L/h/kg	8.994 ± 2.776	6.723 ± 3.009	9.678 ± 1.459
Vz/F/L/kg	78.335 ± 26.335	54.512 ± 23.175*	80.218 ± 19.538
Cmax/mg/L	0.392 ± 0.042	0.904 ± 0.508**	0.218 ± 0.097

Note: *means *p* < 0.05; **means *p* < 0.01.

**TABLE 4:** Comparison of the pharmacokinetic parameters of Rivaroxaban (n = 3).

Parameter	Control	Antibiotic-3days	Antibiotic-7days
AUC(0-t)/mg/L*h	3.342 ± 0.913	3.694 ± 0.369	3.498 ± 0.765
AUC(0-∞)/mg/L*h	4.073 ± 1.365	6.427 ± 3.860*	5.008 ± 1.400
MRT (0-t)/h	8.046 ± 1.233	8.715 ± 1.558	7.246 ± 0.554
t1/2z/h	9.468 ± 2.291	18.307 ± 16.739**	14.846 ± 10.258**
Tmax/h	1.5	0.583 ± 0.144**	0.833 ± 0.144**
CLz/F/L/h/kg	0.699 ± 0.273	0.503 ± 0.256*	0.55 ± 0.166*
Vz/F/L/kg	8.954 ± 1.126	9.305 ± 3.662	10.652 ± 4.879
Cmax/mg/L	0.331 ± 0.005	0.401 ± 0.021*	0.526 ± 0.07**

Note: *means *p* < 0.05; **means *p* < 0.01.

### Gut Dysbiosis Affects the Protein Expression of CYP1A2, CY2C9, CYP3A4, and P-Glycoprotein

**FIGURE 5:**
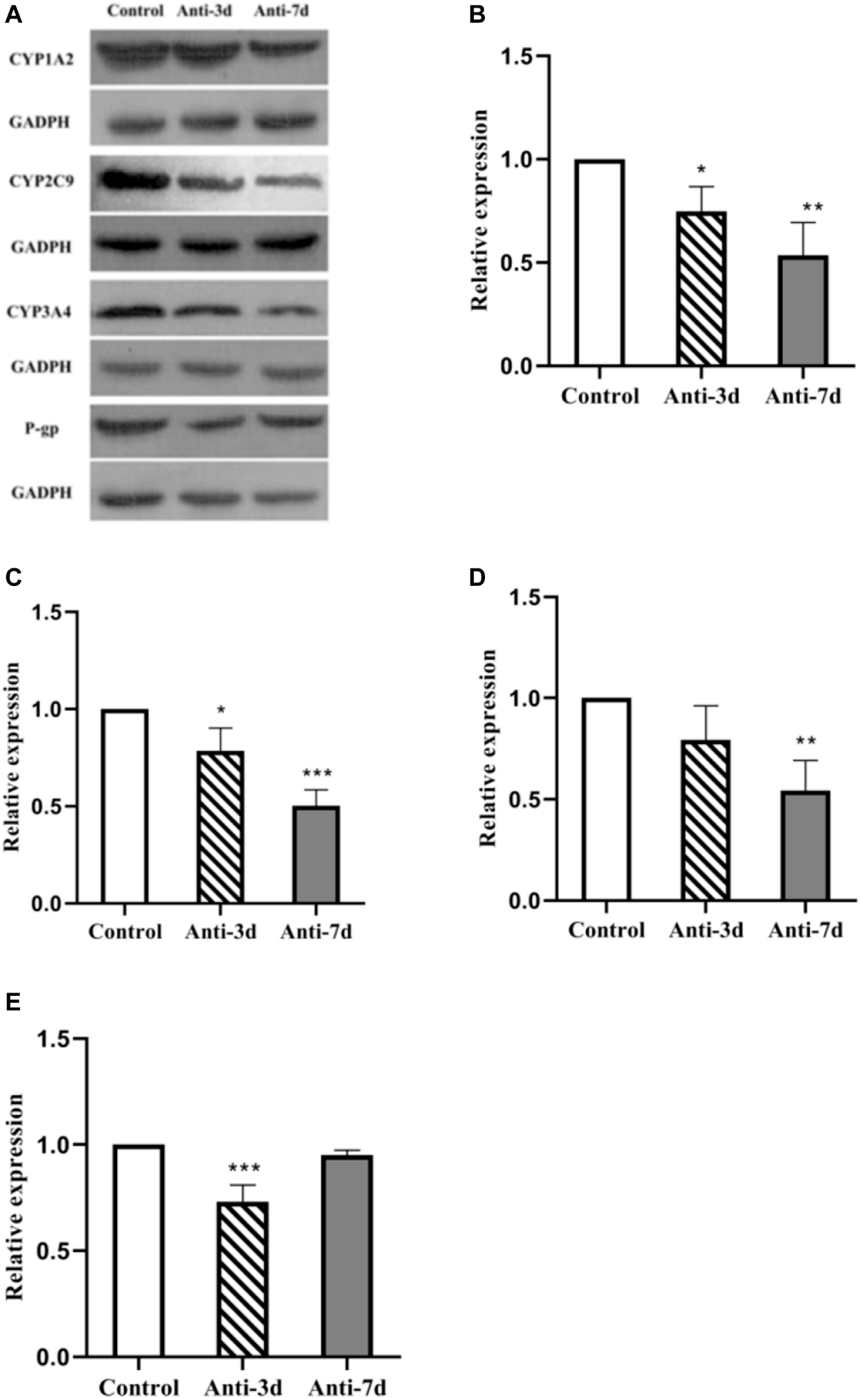
Effect of an intestinal flora imbalance on liver drug enzymes and P-gp protein expression in the terminal ileum. Antibiotics were used to intervene the intestinal flora of rats for 3 and 7 days respectively, and west-blot was used to detect the protein expression levels of liver drug enzymes and P-gp. The level of CYP1A2, CY2C9, CYP3A4, and P-gp protein expression were shown in **(A)**. And the gray analysis results are shown in CYP1A2 **(B)**, CYP2C9 **(C)**, CYP3A4 **(D)** in the liver, and P-gp **(E)** in the ileum. ***p < 0.0001; **p < 0.005; *p < 0.05. The intervention groups were compared with the control group.

Figure 5A shows the level of CYP1A2, CY2C9, CYP3A4, and P-gp protein expression after 3 and 7 days of intervention with amoxicillin, respectively. A gray level analysis of the results was also performed. As shown in Figure 5B, compared with the control group, the level of CYP1A2 protein expression was down-regulated by 25% (*p* = 0.034) in the 3 days amoxicillin treatment group, and was down-regulated by 46% (*p* = 0.002) in the 7 days amoxicillin treatment group. As shown in Figure 5C, compared with the control group, the level of CYP2C9 protein expression was decreased by 20% (*p* = 0.019) in the 3 days amoxicillin treatment group, and down-regulated by 49% (*p* < 0.0001) in the 7 days amoxicillin treatment group. As shown in Figure 5D, compared with the control group, the expression of CYP3A4 was lower (but not statistically significant) in the 3 days amoxicillin treatment group, and was down-regulated by 45% (*p* = 0.005) in the 7 days amoxicillin treatment group. As shown in Figure 5E, compared with the control group, the expression of P-gp decreased by 26% (*p* < 0.001) in the 3 days amoxicillin treatment group, and increased by 22% (*p* = 0.001) in the 7 days amoxicillin treatment group.

### Gut Dysbiosis Affects CYP1A2, P-Glycoprotein, and PXR mRNA Expression

**FIGURE 6:**
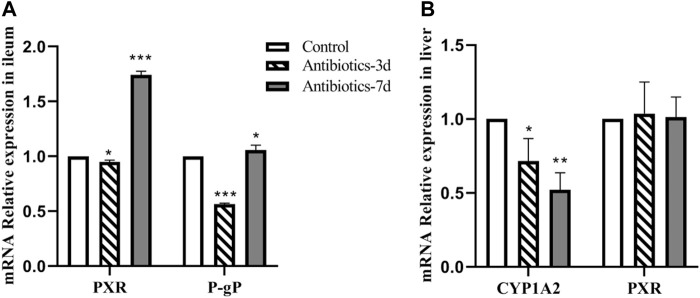
Effect of an intestinal flora imbalance on liver drug enzymes and P-gp mRNA expression in the terminal ileum. Antibiotics were used to intervene the intestinal flora of rats for 3 and 7 days, respectively. RT-PCR was used to detect the level of mRNA expression of PXR and P-gp **(A)** in the ileum, and CYP1A2 and PXR **(B)** in the liver. ***p < 0.0001; **p < 0.005; *p < 0.05. The intervention groups were compared with the control group.

As shown in Figure 6A, compared with the control group, the level of PXR mRNA expression in the ileum of rats in the 3 days amoxicillin treatment group; decreased by 5% (*p* = 0.029), and increased by 74% in the 7 days amoxicillin treatment group (*p* < 0.0001). Similarly, compared with the control group, the level of P-gp mRNA expression in the ileum of rats in the 3 days amoxicillin treatment group; decreased by 44% (*p* < 0.0001), and increased by 6.5% in the 7 days amoxicillin treatment group. These results indicate that the P-gp mRNA expression trend in the ileum was consistent with that of PXR mRNA. As shown in Figure 6B, compared with the control group, CYP1A2 expression in the liver of rats in the 3 days amoxicillin treatment group was decreased by 28% (*p* = 0.02), and by 48% in the 7 days amoxicillin treatment group (*p* = 0.002); however, there was no statistical difference in the level of PXR expression in the liver of each group.

## Discussion

Two important problems faced by the clinical application of oral anticoagulants are: insufficient anticoagulation and excessive anticoagulation. If anticoagulation is insufficient, there is an increased risk of thrombosis. In contrast, over anticoagulation will increase the risk of bleeding. Therefore, oral anticoagulants play an important role in anticoagulant therapy; however, its ideal effect is affected by many factors. This study is the first to investigate the impact of changes in the gut microbiota on the efficacy of oral anticoagulants. In our rat model of gut dysbiosis, compared with the control group, the bioavailability of warfarin and rivaroxaban increased in the antibiotic-treated group. In contrast, the bioavailability of dabigatran increased in the 3 days amoxicillin treatment group but decreased significantly in the 7 days amoxicillin treatment group. Therefore, altered microbial microenvironments may affect the efficacy of oral anticoagulants by influencing their pharmacokinetic processes. Our investigation into the possible internal mechanism of the above phenomenon revealed that the level of CYP1A2, CYP2C9, and CYP3A4 expression in the liver of rats with a gut dysbiosis (groups that were amoxicillin-treated for 3 and 7 days) was significantly decreased, with the most significant decrease in the 7 days amoxicillin treatment group. The expression of P-glycoprotein in the ileum was significantly decreased in the 3 days amoxicillin treatment group but increased in the 7 days amoxicillin treatment group. It is speculated that the gut microbiota can influence the metabolism of oral anticoagulants by regulating the expression of hepatic drug enzymes and transporter proteins, thereby affecting their pharmacokinetic processes. In addition, our results suggest that there may be some differences in the influence of gut microbiota on the expression of hepatic drug enzymes and transporter proteins. With the extension of antibiotic treatment time, the influence of gut microbiota changes on liver drug enzyme expression exhibited a stable inhibitory effect, whereas the influence on transporter expression was relatively unstable: an inhibitory effect was observed in the 3 days amoxicillin treatment group whereas the 7 days amoxicillin treatment group promoted its expression.

Warfarin is primarily metabolized into inactive metabolites by the liver enzymes CYP2C9, CYP1A2, and CYP3A, and excreted through the kidney (Shaik et al., 2016). In this study, it was found that changes in the gut microbiota significantly reduced the expression of the liver drug enzymes, CYP1A2, CYP2C9, and CYP3A4, which reduced the metabolism of warfarin in the liver. Moreover, the clearance rate (CLz/F) of warfarin was reduced, the mean residence time (MRT) in the body was prolonged, and the bioavailability increased. Among these, the bioavailability of warfarin in the 7 days amoxicillin treatment group increased by 115%, the peak concentration (Cmax) increased by 74%, and the peak time was reduced from 3 to 1 h. Moreover, the changes in the gut microbiota also extended the half-life of warfarin. In particular, the half-life of warfarin in the 7 days amoxicillin treatment group doubled compared with the control group (15.468 ± 1.47 h, 32.454 ± 1.751 h; *p* < 0.05). Therefore, the INR should be closely monitored for patients taking oral anticoagulants who are clinically receiving antibiotics or whose physiological status may affect the gut microbiota. In addition, a timely dosage adjustment should be made to avoid the occurrence of adverse reactions, such as bleeding.

Dabigatran is mainly affected *in vivo* by the drug efflux pump, P-glycoprotein, which is the product of the multi-drug resistance 1 gene^10^. In this study, it was found that changes in the gut microbiota had the same effect on the expression of ABCB1 and PXR mRNA in the ileum. This finding suggests similar trends of P-GP and PXR expression in the rats with a gut dysbiosis, indicating that the intestinal flora may affect the expression of P-GP protein by regulating the nuclear receptor PXR in the intestinal tract. This effect is consistent with the changes in the pharmacokinetic parameters of dabigatran. In the 3 days amoxicillin treatment group, the expression of P-gp was inhibited, resulting in a decrease in dabigatran efflux and the greatest increase in bioavailability (AUC increased by 53%, Cmax increased by 130%) (*p* < 0.05); while the expression of P-gp increased and the bioavailability of dabigatran decreased in the 7 days amoxicillin treatment group (AUC decreased by 11%, Cmax decreased by 44%). In addition, the study of Yoo et al. (Lavrijsen et al., 1995) found that the gut microbiota may play a role in the conversion process of propharmaceutical drugs into active substances. As a precursor of dabigatran, the activity conversion process of dabigatrine may also be affected by gut microbiota; however, the specific situation requires further study.

Rivaroxaban is primarily metabolized by CYP1A2 and CYP3A4 in the body (Gelosa et al., 2018), and it is also a substrate of the P-glycoprotein ^12^; thus, its bioavailability is affected by the combined effects of liver drug enzymes and P-glycoprotein. In this study, in the 3 days amoxicillin treatment group, the expression of CYP1A2, CYP3A4, and P-glycoprotein was decreased, which promoted the maximum increase in the bioavailability of rivaroxaban (AUC increased by 10%). In the 7 days amoxicillin treatment group, although changes in part of the flora increased its outward transport, the decreased expression of liver drug enzymes had a stronger inhibitory effect on its metabolic process. After the two were superimposed, the bioavailability of rivaroxaban continued to increase (AUC increased by 4%). At the same time, the change in the gut microbiota also had a certain effect on the half-life of dabigatran and rivaroxaban. Therefore, changes in the gut microbiota may also affect the *in vivo* disposal of new oral anticoagulants (e.g., dabigatrine and rivaroxaban), which may represent one of the reasons for their individualized differentiation.

It has been well-established that the level of liver drug enzyme expression is regulated by a variety of nuclear receptors, including the constitutive androstenoid receptor (CAR), progesterone X receptor (PXR), Arnesoid X receptor (FXR), and aryl hydrocarbon receptor (AHR) (Gadaleta et al., 2015; Swanson, 2015). Among these, PXR is particularly important for the disposal of exogenous substances in the body. This is because PXR has a large and flexible ligand binding domain, which enables it to respond to a variety of exogenous drugs (Wallace and Redinbo, 2013). Studies have shown that changes in the gut microbiota can affect the expression of nuclear receptors (Lundin et al., 2008; Björkholm et al., 2009; Selwyn et al., 2015); however, it remains controversial whether changes in the gut microbiota affect the expression of liver drug enzymes through direct or indirect intervention through nuclear receptors. In the study by Selwyn et al. (2016), the expression of liver drug enzymes was considered to be dependent on the activation of respective PXR or FXR ligands. In contrast, the study by Claus et al. (2011) found that the expression of AHR, CAR, FXR, PXR, and RXRa in GF, CV, and C3H/Orl mice was similar, whereas their regulation of CYP450 gene expression (CYP2C29, CYP3A11, and CYP8B1) differed. These results are consistent with our finding that changes in the gut microbiota did not affect the level of PXR expression, but significantly reduced the level of CYP1A2 expression. This suggests that the influence of the gut microbiota on the regulation of liver drug enzyme expression is not a closed response pattern.

In our study, amoxicillin was selected as a model antibiotic to construct a rat model of gut dysbiosis. Studies have shown that amoxicillin does not affect the metabolic activity of P450 liver drug enzymes (e.g., CYP2C9 and CYP3A4) (Niwa et al., 2016), and no studies have demonstrated the ability of amoxicillin to affect P-glycoprotein activity. At the same time, oral anticoagulants were administered orally after the excretion of amoxicillin to reduce the influence of factors other than the gut microbiota on the *in vivo* disposal of oral anticoagulants and to ensure that an imbalance of the gut microbiota played a major role in the pharmacokinetic parameter changes of the oral anticoagulants. The 16S rRNA high-throughput sequencing analysis was performed in rat feces before and after amoxicillin intervention. Similar to the reports of other studies (Stewardson et al., 2015; Korpela et al., 2016; Kuno et al., 2016), the gut microbiota of the rats changed its structure and composition after exposure to antibiotics. There was a significant decrease in the richness and diversity of the gut microbiota, and there was also a significant change in the dominant flora following gut dysbiosis, suggesting the successful construction of the gut dysbiosis model.

The structure of the human gut microbiome begins during the neonatal stage and changes as the host grows. During this process, the composition of the gut microbiota is influenced by diet, gender, geographic location, and ethnicity (Scott et al., 2013; Prieto et al., 2018). Therefore, the gut microbial microenvironment of patients receiving oral anticoagulants differs between individuals. The results of this study suggest that differences in the gut microbiota may lead to changes in the pharmacokinetic parameters of oral anticoagulants, which may represent a novel mechanism that leads to individual differences in oral anticoagulants. Previous studies (Matuskova et al., 2014) have shown that patients can take probiotics daily, which can both suppress the production of harmful bacteria in the intestine, as well as provide a favorable environment for the growth of beneficial bacteria in the intestine to create a healthy intestine, which may be beneficial for improving the individual differences in oral anticoagulants.

There are still some limitations of our study. First, although this study confirmed that the gut microbiota can affect the bioavailability of oral anticoagulants by changing the expression of related metabolic enzymes and transporters, it is unclear which specific gut microbiota or its metabolites are involved in this process. In addition, as we gain further knowledge regarding how the gut flora promotes drug metabolism, we must develop more advanced experimental methods, including fecal transplantation, to more fully determine its overall impact on patient response.

## Conclusion

Our study represents a novel mechanism for influencing individual differences in oral anticoagulants. Changes in the intestinal flora can affect the expression of liver drug enzymes and P-gp to a certain extent, and subsequently affect the transport and metabolism of oral anticoagulants (e.g., warfarin, dabigatracin, and rivaroxaban), leading to differences in the treatment efficacy of oral anticoagulants, and even cerebral hemorrhage, cerebral infarction and other serious adverse event. Thus, individual differences in the treatment efficacy of oral anticoagulants can be improved by taking probiotics or specific beneficial bacteria.

## Data Availability

The data presented in the study are deposited in the SRA repository, accession number: PRJNA807515 (https://www.ncbi.nlm.nih.gov/sra/PRJNA807515).
